# Editorial Note: Additive manufacturing process selection for automotive industry using Pythagorean fuzzy CRITIC EDAS

**DOI:** 10.1371/journal.pone.0357931

**Published:** 2026-09-09

**Authors:** The PLOS One Editors

The *PLOS One* Editors issue this Editorial Note to inform readers that works cited in this article [1] as References 53, 55, and 89 were retracted after the article’s publication date. PLOS considers that these references are not crucial in supporting the research or conclusions reported in [1].
